# Brain Mitochondrial Bioenergetics in Genetic Neurodevelopmental Disorders: Focus on Down, Rett and Fragile X Syndromes

**DOI:** 10.3390/ijms241512488

**Published:** 2023-08-06

**Authors:** Daniela Valenti, Rosa Anna Vacca

**Affiliations:** Institute of Biomembranes, Bioenergetics and Molecular Biotechnologies (IBIOM), National Research Council (CNR), Via G. Amendola 122/O, 70126 Bari, Italy

**Keywords:** brain mitochondrial bioenergetics, neurogenesis, neuroplasticity, genetic neurodevelopmental disorders, Down syndrome, Rett syndrome, Fragile X syndrome

## Abstract

Mitochondria, far beyond their prominent role as cellular powerhouses, are complex cellular organelles active as central metabolic hubs that are capable of integrating and controlling several signaling pathways essential for neurological processes, including neurogenesis and neuroplasticity. On the other hand, mitochondria are themselves regulated from a series of signaling proteins to achieve the best efficiency in producing energy, in establishing a network and in performing their own de novo synthesis or clearance. Dysfunctions in signaling processes that control mitochondrial biogenesis, dynamics and bioenergetics are increasingly associated with impairment in brain development and involved in a wide variety of neurodevelopmental disorders. Here, we review recent evidence proving the emerging role of mitochondria as master regulators of brain bioenergetics, highlighting their control skills in brain neurodevelopment and cognition. We analyze, from a mechanistic point of view, mitochondrial bioenergetic dysfunction as causally interrelated to the origins of typical genetic intellectual disability-related neurodevelopmental disorders, such as Down, Rett and Fragile X syndromes. Finally, we discuss whether mitochondria can become therapeutic targets to improve brain development and function from a holistic perspective.

## 1. Introduction

Traditionally known as the main cell producers of energy currency in the form of ATP, mitochondria are the most studied cellular organelles due to their structural and functional complexity and their plethora of crucial tasks, such as the regulation of calcium homeostasis, the maintenance of redox balance, the generation of bioactive metabolites, the initiation of apoptosis and the regulation of epigenetics, to cite only a few [1,2].

Mitochondria are also considered important metabolic hubs for their peculiar ability to integrate several signaling networks and metabolic pathways to control cellular functions. However, they are themselves regulated from several cellular effectors, including specific kinases and transcription factors, to reach the best efficiency in producing energy in the form of ATP through the oxidative phosphorylation (OXPHOS) process, through movement inside and outside the cells to reach specific districts, and through performing their own de novo synthesis or clearance. These complex processes, named mitochondrial bioenergetics, dynamics, biogenesis and mitophagy, always take place in response to cellular energy demands [3,4,5]. For these reasons, mitochondria numbers and locations depend on tissue/organ metabolic requests.

The brain is the organ with the highest energy requirement, consuming up to 20% of all bodily energy, which takes place 10 times faster than the rest of the body per gram [6,7]. Recently, Bülow and colleagues [8] have estimated that the human brain burns five orders of magnitude more energy than that produced by the sun per unit of mass, reaching a peak of oxygen and glucose consumption to produce ATP around five years of age. Indeed, a child’s brain consumes about 50% of the body’s energy cost that it needs for development and plasticity [9]. Neurons are responsible for a large part of the energy consumption of the brain that is necessary for the maintenance of the dynamics of the actin cytoskeleton and the complex formation of synapses. Astrocytes also sustain the bulk of the metabolic weight in order to control the interstitial fluid composition, to store energy in the form of glycogen, to supply neurons with fuel sources and metabolites for biosynthesis and to recycle neurotransmitters, oxidation products and other metabolic waste products [9,10].

Several factors influence brain development during the embryological and neonatal critical window, when genetic programming combined with environmental input influence brain modeling and produce neural circuits that mediate a highly complex set of behaviors. A crucial event of neurodevelopment is the process of neurogenesis, which includes the proliferation, migration and differentiation of neural cells, neuroplasticity, the formation of neuronal connectivity and synaptic rearrangement and, ultimately, functional maturation of the neurological pathways responsible for the brain’s ability to learn and to develop memory and social and emotional skills [11,12]. In all these processes, mitochondria play a central regulatory role (see Section 2, Section 3 and Section 4 for insight).

Abnormalities in the physiological processes of neurodevelopment that occur during the prenatal/neonatal critical window, often due to alterations in genetic, epigenetic and environmental factors, induce typical disorders described as neurodevelopmental diseases (NDDs), which are characterized by impairments in cognitive, communicative, behavior and motor skills [13,14]. Numerous studies and emerging data have revealed a correlation between dysfunctional mitochondrial bioenergetics and NDDs (for references, see reviews [8,9,15]).

Common findings have associated disorders with different genetic causes, such as monogenic defects being responsible for syndromes such as Rett and Fragile X and aneuploidy being associated with chromosomal copy number variations that are responsible for Down syndrome. Some of these common altered features can essentially be summarized by lower brain ATP levels, which are associated with higher lactate levels, indicating a switch from OXPHOS to glycolysis as an energy source, and in the abnormal expression and activity of mitochondrial respiratory chain (MRC) complexes, associated with an increase in free radical species produced by the dysfunctional MRC [16,17,18,19,20,21,22].

This review will address the emerging role of mitochondrial bioenergetics in brain development, analyze how mitochondria bioenergetic dysfunction is causally interrelated to the origins of neurodevelopmental disorders and discuss whether targeting mitochondria can become a therapeutic strategy to improve brain development and function.

## 2. Mitochondrial Energy Metabolism in the Brain: Role of Neurons and Glia

The human nervous system is equipped with a wide range of cell types, principally represented by neurons and glial cells, as well as immune and vascular endothelial cells. Glia comprise a heterogeneous group of cell types including astrocytes, oligodendrocytes, microglia and ependymal cells. The overall number of and reciprocal proportion ratios between the nervous cells remains an open question, but there is evidence that the neuron-to-glia ratio approaches one in the human brain [23]. Glial cells are responsible for maintaining energy homeostasis of the nervous system [24]. Although glia are not excitable cells and do not directly receive and process synaptic inputs, they play an active and crucial role in regulating and maintaining synaptic structures and synaptic transmission [25,26]. Different glial cell types possess distinct metabolic and mitochondrial properties from neurons and are differentiated from one another. Microglia are macrophage-derived glial cells that perform crucial immunological and phagocytic functions within the central nervous system (CNS) [27]. Under physiological conditions, microglia “police” the CNS by responding to a variety of host-derived or pathogenic signals and assuming different phenotypic profiles. Differences have been identified between “quiescent” and “reactive” microglia [28]. After transitioning from a quiescent to a reactive state, OXPHOS in microglia is reduced, and the production of aerobic glycolysis and reactive oxygen species (ROS) is increased [29].

Oligodendrocytes provide electrical insulation to axons through the formation of the myelin sheath, with the maintenance of myelin being an energy-intensive process [30].

Astrocytes are very abundant glial components in the nervous system. They are strictly incorporated into neuronal circuits that are directly involved in maintaining nervous energy homeostasis.

Astrocytes and neurons exhibit a different metabolic profile: neurons metabolize glucose aerobically to produce ATP through mitochondrial OXPHOS [31], whereas glucose enters astrocytes preferentially routed in the glycolytic pathway for the generation of ATP, pyruvate and lactate [32,33]. Astrocytes and neurons also show different regulation processes of the glycolytic pathway and differ in the organization of their MRC. Unlike neurons, astrocytes show a reduced supramolecular assembly of MRC complexes, by which mitochondrial ROS are overproduced (for refs., see [34,35]).

Astrocytes are particularly important regulators of oxidative homeostasis during development [36]. These glial cells contain an important part of antioxidant enzymes [37] within the brain as well as cellular sources of ROS, which largely affect redox balance and neuronal function during neurodevelopment [38].

Glucose is the preferential energy source for the adult brain. However, under particular conditions, the brain adapts to consume other energy substrates, such as ketone bodies in the development phase or during fasting [39,40] or lactate during the physical effort [41]. Therefore, some intermediates of glycolytic metabolism such as lactate, pyruvate or acetate can be oxidized by the brain to meet its energy needs [42,43]. In particular, several studies have highlighted the importance of lactate as an energy substrate for the brain [44,45,46,47,48]. Specifically, results from in vitro and in vivo studies have demonstrated that lactate supports neuronal activity during glucose deprivation [49,50]. The astrocyte–neuron lactate shuttle hypothesis suggests that astrocyte-derived L-lactate is transported into neurons via monocarboxylate transporters and used as a preferential energy substrate with respect to glucose [51]. Despite its relatively low level in the brain compared to other organs such as muscle and the liver, glycogen comprises the brain’s energy store. It is a useful form of storage for glucose, which can be rapidly metabolized and, unlike fatty acids, can produce ATP under anaerobic conditions. Interestingly, at the cellular level, glycogen is localized almost exclusively in astrocytes of the adult brain [52,53], demonstrating that glycogen constitutes a specific feature of astrocytic glucose metabolism requiring metabolic interactions between astrocytes and neurons. Glycogen mobilization may satisfy the astrocyte’s own metabolic needs [54,55]; in addition, lactate released into the extracellular space as a result of degradation of glycogen in astrocytes [55,56] can feed the energy requirement of neurons.

In light of recent evidence, astrocytes have emerged as key regulators of brain energy metabolism and active players in the production, refueling, utilization and storage of brain energy [35]. We are progressively changing our point of view of brain bioenergetics from a “neuro-centric” vision to a more complex picture involving a deep astrocyte–neuron metabolic cooperation [57].

## 3. Mitochondrial Bioenergetics in the Regulation of Neurogenesis

**Figure 1:**
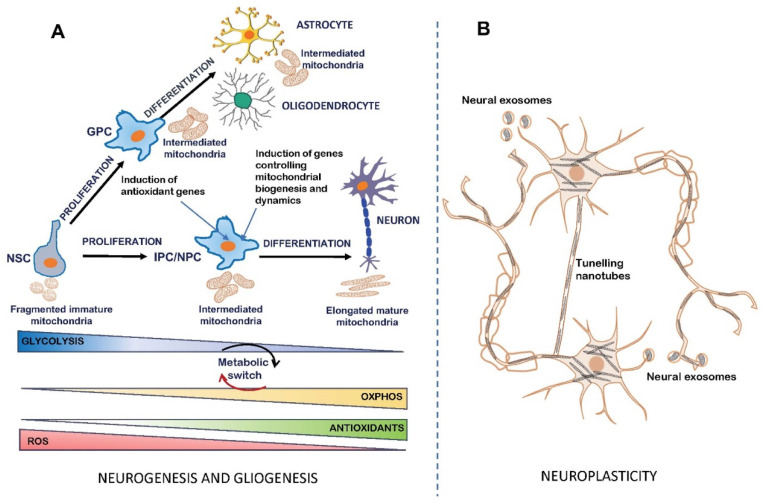
Schematic picture of mitochondria involvement in neurogenesis/gliogenesis and neuroplasticity. During neurogenesis and gliogenesis (**A**), neural stem cells (NSCs) can self-renew and proliferate, producing glia progenitor cells (GPCs) and neuron progenitor cells (NPCs), which differentiate into astrocytes and oligodendrocytes as well as neurons, respectively. NSCs and both GPCs and NPCs have immature and poor oxidative phosphorylating mitochondria (levels of OXPHOS are indicated as yellow increasing box) with a prevalent glycolytic metabolism (levels of glycolysis are indicated as blue decreasing box), accompanied by a high production of reactive oxygen species (ROS) (indicated as red decreasing box). Because of the induction of genes controlling mitochondrial biogenesis and dynamics and the antioxidant system, mitochondria activation, maturation and elongation occur, with a consequent metabolic switch from glycolysis towards OXPHOS and a decrease in ROS accumulation due to the increase in antioxidants (levels of antioxidants are indicated as green increasing box). These events induce NPCs to differentiate into neurons. Conversely, the metabolic switch does not occur during GPC differentiation into the glial cells, astrocytes and oligodendrocytes, maintaining a prevalent glycolytic metabolism, high ROS levels and poor mitochondrial OXPHOS metabolism (for references, see [78]). During neuroplasticity (**B**), mitochondria play active roles in regulating the outgrowth of axons and dendrites by movement in either the anterograde or retrograde direction to produce ATP in specific neuronal districts. As depicted in the scheme, the mitochondrial translocation can occur inside the neurons or outside neuron boundaries through tunneling nanotubes or extracellular vesicles, such as exosomes (for references, see [78]).

Neurogenesis is the process of transformation of neural stem cells into actively proliferating progenitor cells that, in the final stage, differentiate into mature new neurons [58,59]. Neural stem cells (NSCs) are a population of pluripotent cells in the nervous system that give rise, through the intermediate neuron progenitor cells (NPCs) and glia progenitor cells (GPCs), to neurons and glial cells, including oligodendrocytes and astrocytes [60] (Figure 1). Importantly, mammalian NSCs may be present not only during embryonic brain development [61], but also in the neonatal and adult brain [62,63].

For a long time, it was believed that the neural stem cell pool was almost totally depleted during the perinatal phase, leading to an arrest of neurogenesis in the early stages of neonatal life. Though this is certainly true for many brain regions, neurogenesis persists into adulthood in specific areas of the mammalian brain: the subependymal zone of the lateral ventricle (SEZ) of the olfactory bulb and the subgranular zone (SGZ) of the dentate gyrus of the hippocampus [64,65,66]. However, the extent to which neurogenesis occurs in human adulthood still remains an object of discussion [67,68,69]. New evidence reports that neurogenesis may also be supported outside the classical neurogenic niches in other brain regions of the adult mammalian brain, including the hypothalamus, striatum, substantia nigra, cortex and amygdala (for references, see [70]).

In the process of generating neurons, the programming of energy metabolism undergoes critical changes. The processes of neuronal proliferation/differentiation and neuronal activity require a significant energy expenditure. Mitochondria are important for the regulation of NPCs during the process of neurogenesis (see Figure 1A). Neural stem cells rely on glycolysis to meet their energy demands [71]. Apart from their glycolytic profile, NSCs have immature fragmented mitochondria characterized by a spherical ultrastructure with poorly matured cristae and few copies of mitochondrial DNA [72]. As NSCs differentiate into intermediate progenitor cells (IPCs) and neural progenitor cells (NPSs), their energy metabolism relies in part on OXPHOS, reducing the contribution from glycolytic metabolism and lactate production [73,74,75,76]. Once mature neurons form, they rely completely on OXPHOS-dependent energy metabolism to perform neuronal activities [73,77]. During gliogenesis, the process by which NSCs differentiate into astrocytes and oligodendrocytes through the glia progenitor cells (GPCs), mitochondria do not complete the maturation processes. For example, MRC is not organized into supramolecular complexes, and they remain poor energy producers [78].

Zheng et al. investigated the metabolism switch from anaerobic glycolysis in neural progenitor cells to OXPHOS in mature neurons, indicating that this metabolic shift is essential for neuronal differentiation and matured neuron formation, and it is a critical process in ensuring energy support for neurogenesis [79]. The starting point of the metabolic switch occurs through the gene expression induction of PGC-1α, the master regulator of mitochondrial biogenesis [80], and an increase in the synthesis of MRC complexes’ subunits [65]. Mitochondrial mass increases to sustain the MRC machinery, in association with OXPHOS required for neuronal differentiation [81,82]. During neurogenesis, in order to acquire an OXPHOS metabolic profile, mitochondria increase in number and undergo structural changes to assume an elongated morphology in neurons [79,83,84,85]. Mitochondrial dynamics also regulate the activation of neurogenesis in NSC-derived NPCs [86].

Disturbances in mitochondrial biogenesis and OXPHOS during brain development prevent the metabolic switch from glycolysis to OXPHOS that is strictly required to initiate the process of neurogenesis (for refs., see reviews [73,84,87,88]). Defective OXPHOS bioenergetics or dysfunctions in the regulation of mitochondrial biogenesis and dynamics could compromise neurogenesis and the physiological establishment of neuronal function.

## 4. Critical Roles of Brain Mitochondria as Master Regulators of Neuronal Plasticity and Central Hubs of Synaptic Modulation

The maintenance and regulation of cellular energy metabolism is a critical challenge for the nervous system. The brain’s metabolic balance is finely controlled by the involvement of a close metabolic integration between neurons and glia, which together form an integrated metabolic unit to meet energy needs of neural circuits [32,48,89]. The metabolic cost of performing and sustaining basal neural functions is enormously high and requires functioning mitochondria. For instance, a single neuron can contain thousands of mitochondria [90], whose function is very crucial to sustain neuronal physiology. In the brain, mitochondria are essential for cell growth [86], neurotransmission [91], the maintenance of cell membrane ion gradients [92] and synaptic pruning [93]. Mitochondrial bioenergetics critically sustain numerous ATP-dependent processes that allow neurons to function and respond adaptively to environmental challenges in the process known as neuroplasticity [94,95]. Neuroplasticity is defined as the ability of the brain to undergo a series of adaptive changes in the structure and function of nervous cells in response to physiological or pathological perturbations [96]. Examples of neuroplasticity include the growth of axons or dendrites, the formation of synapses, the consolidation of synapses in response to repeated nerve impulses and neurogenesis. In addition to neurons, glial cells play important roles in neuroplasticity by producing soluble and surface-bound factors that impact neurite outgrowth, synaptic activity and cell survival [25,97].

Two significant examples of neuroplasticity are long-term potentiation (LTP) and long-term depression (LTD), present in neuronal synapses in response to rapid repeated stimulation [98]. LTP is a form of activity-dependent plasticity resulting in a persistent enhancement of synaptic transmission, whereas LTD consists of a decrease in the efficacy of a synapse as a result of a particular type of stimulation. LTP is also considered to be the cellular mechanism of learning and memory [99,100]. Several studies have documented that changes in mitochondria occur during synaptic activation and LTP [100]. For example, during LTP, mitochondrial energy production undergoes changes [101], mitochondrial calcium pump activity increases [102], and mitochondrial gene expression results are enhanced [103].

Mitochondria are highly mobile and move rapidly within and between subcellular compartments of the neurons involved in neuroplasticity [104]. Mitochondria are mostly located along the length of axons and in both the pre- and post-synaptic terminals, providing the energy necessary for the activity of these specialized neuronal compartments. At the dendritic level, they are principally present in dendritic extensions and less frequently associated with spines [105,106].

Mitochondria undergo highly coordinated processes of fission and fusion [107], responding to the activation of neurotransmitters and growth factor receptors [108].

Local translation in neurons is fueled by mitochondria that support neuronal plasticity in dendrites and axons, with the mitochondrial compartments able to function as a local energy supply for synaptic translation [109]. Mitochondrial biogenesis occurs locally to maintain the functional activity of mitochondria in axons and dendrites, indicating that local mitochondrial protein production plays an essential role in synaptic functions [110].

The biogenesis of mitochondria and their movement along the axon is particularly important because the axon grows much faster and longer than the dendrites, therefore requiring more energy to sustain its rapid growth. As synapses are formed, the number of mitochondria increases, and synaptic activity influences the distribution of mitochondria along the length of the dendrites and axons, in the presynaptic terminals and at the base of the dendritic spines [111]. Synaptic activity affects the positioning of mitochondria at the base of dendritic spines, where they play an important role in the structural plasticity of the spines [91].

The biogenesis of neuronal mitochondria usually takes place in the cell body, from which the mitochondria form a dynamic network that is distributed in the various compartments of neurons [112]. However, axonal mitochondria are much more dynamic than the mitochondria present in dendrites [113,114]. They are highly mobile, and their movement can occur in both anterograde and retrograde directions [115]. Recent studies have revealed that the dynamic nature of mitochondria can go beyond neuron boundaries [4]. Indeed, mitochondria can translocate between neurons through neural vesicles called exosomes or nano-tunnels; this accounts for the further signaling role of mitochondria as mediators of communication between neurons (see Figure 1B). 

As far as transport of axonal mitochondria, they are carried out to the synapse by the motor proteins kinesin and dynein, respectively, which carry their mitochondrial cargo along microtubule tracks [116]. Mitochondria in neurons can be more present and concentrated in neuronal districts with major metabolic energy needs, such as growth cones and/or pre- and post-synaptic spaces [117]. The transport of mitochondria along the neurite is a highly regulated process, and the activity of mitochondria in the synapses allows for the integration of different signals; thus, mitochondria are key players in the modulation of synaptic stimulation [118]. Mitochondria clustered at the synaptic level constitute a discrete mitochondrial pool distinct from non-synaptic neuronal mitochondria, exhibiting different morphological and proteomic features and an increased vulnerability to oxidative damage (for references, see [118]).

Different metabolic energy requirements are linked to the heterogeneity among subcellular compartments in different brain districts [119], indicating that neuronal mitochondria may be subjected to different processes of regulation based on the neuronal compartment and depending on the brain area.

## 5. Defective Brain Mitochondrial Bioenergetics in Intellectual Disability Diseases: The Case of Particular Genetic Neurodevelopmental Diseases

**Figure 2:**
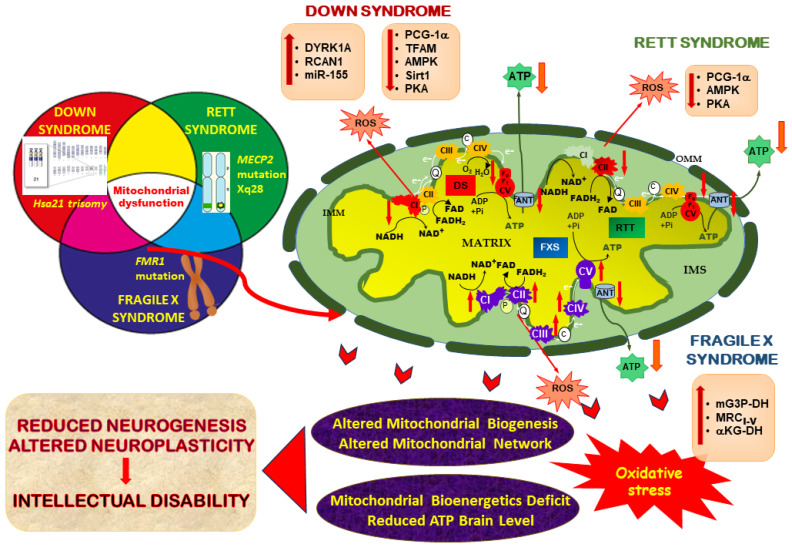
Mitochondrial bioenergetic dysfunction as a common factor in neurodevelopmental diseases. The cases of Down syndrome (DS), Rett syndrome (RTT) and Fragile X syndrome (FXS) are summarized. As a result of altered signaling pathways controlling mitochondrial function, selective mitochondrial respiratory chain complexes are impaired. Down regulation of the complexes I and V (in red, down arrow) occurs in the case of DS; down regulation of the complexes II and V (in red) occurs in the case of RTT; and a general upregulation of all MRC complexes (depicted in purple, up arrow) occurs in the case of FXS. In all cases, the MRC impairments result in a reduction in ATP production through OXPHOS and an increase in mitochondrial ROS, with a consequent impairment in mitochondrial bioenergetics, biogenesis and dynamics and oxidative stress. These mitochondrial dysfunctions play a central role in neurogenesis and neuroplasticity defects that lead to intellectual disability. Abbreviations: AMPK, AMP-activated protein kinase; ANT, adenine nucleotide translocator; αKG-DH, alpha ketoglutarate dehydrogenase; DYR1A, dual-specificity tyrosine phosphorylation-regulated kinase 1A; mG3P-DH, mitochondrial glycerol 3 phosphate dehydrogenase; miR-155, microRNA 155; PGC-1α, peroxisome proliferator-activated receptor gamma coactivator-1alpha; PKA, protein kinase A RCAN1, regulator of calcineurin 1; Sirt1, sirtuin 1; TFAM, mitochondrial transcription factor A; IMM, inner-membrane space; IMS, inter-membrane space; OMM: outer-mitochondrial membrane.

In this section, we focus on specific genetic neurodevelopmental diseases, including Down, Rett and Fragile X syndromes, since these are the most typical genetic syndromic conditions leading to intellectual disability. For these diseases, brain energy deficits and defective mitochondrial bioenergetics were reported to be linked to their etiopathology, playing a major role in the development of the neuropathological symptoms (Figure 2).

### 5.1. Down Syndrome

Down syndrome (DS, OMIM #190685) is a genetic neurodevelopmental disease that is due to a typical chromosomal aneuploidy, the human chromosome 21 (Hsa21) trisomy, caused by the presence of a third copy of the Hsa21 in whole or in part. The overexpression of Hsa21 genes and microRNAs and the ensuing effects on the entire transcriptome [120,121] result in distinctive clinical signs such as hypotony at birth, typical facial traits, congenital heart, gastrointestinal and multiple organ defects, an increased risk of a series of chronic illness conditions and early onset of Alzheimer-like dementia and aging.

DS is also considered to be the most significant genetic disorder leading to neurodevelopment abnormalities, neurobehavioral disorders, alteration in brain development and developmental delay (for refs., see reviews [18,122]). Therefore, intellectual disability, from mild to severe, is the most striking hallmark of the DS phenotype. From the fetal life, mitochondrial dysfunctions are reported to be widely present in a variety of cells with Hsa21 trisomy, including neurons and astrocytes [16,17], and these also occur early in brain tissues of mouse models of DS [123]. Many research groups, including ours, have extensively studied the molecular bases of the mitochondrial phenotype in DS and validated the impact of the neural bioenergetic deficit on neurogenesis and neuroplasticity, as well the early neurodegeneration processes in DS, as widely reviewed in [17,18,124,125,126,127,128,129]. A dosage imbalance arising from Hsa21 genes (e.g., *CBS*, *RCAN1*, *DSCAM*, *DYRK1A*, *NRIP1*) and microRNAs (e.g., let-7c and miR-155) impairs key regulatory signaling pathways controlling mitochondrial functions (e.g., the cAMP/PKA [130,131]), the co-activator PGC-1α/PPARGC1A [123,132,133], Sirt1/AMPK [132], Drp1/Mnf2/Opa1 [134], rapamycin mTOR [135,136] and insulin pathways [137]. These signaling imbalances result in the reduced expression and function of protein products of OXPHOS machinery, inducing impairment in mitochondrial ATP production, decreased respiratory capacity, impaired ability to generate mitochondrial membrane potential and ROS overproduction. Alterations in mitochondrial biogenesis and fusion/fission dynamics also lead to bioenergetics defects, network disruption and mitophagy in DS [124,127,134].

Disturbances in mitochondrial biogenesis and in OXPHOS during brain development prevent the metabolic shift from glycolysis to OXPHOS, which is necessary to initiate the neurogenesis process (as described in Section 3). Indeed, glycolysis and the levels of brain lactate remain high in DS to compensate for the mitochondrial OXPHOS deficit [134,138], and it might be that the glycolytic environment can change the fate of neural progenitor differentiation negatively, affecting neurogenesis and favoring astrocyte formation [18]. Recently, this hypothesis has been confirmed in neonatal astrocytes from a Ts65Dn mouse model of DS [139] and in the trisomic neural progenitor cells (NPCs) obtained from Hsa21 trisomic-induced pluripontent stem cells (iPSCs). NPCs display early mitochondrial dysfunction and an abnormal mitochondrial network during differentiation with respect to euploid cells and show greater potential in producing glial-like cells, which leads to a failing energy metabolism transition [125,140]. Actually, a reduction in hippocampal neuronal proliferation potency, maturation and connectivity as well dendritic alterations have been also reported in the neonatal period in Ts65Dn mouse models of DS [141,142,143]. Since a functional mitochondrial network is crucial for district-dependent energy production in the axonogenesis and synaptogenesis processes (see above, Section 4 for insight), alterations in mitochondrial bioenergetics and dynamics could be crucial for dictating the impairment in the overall neuroplasticity process in DS.

Moreover, dysfunctional mitochondria, and in particular defects in mitochondrial respiratory chain complex I, overproduce superoxide anion (O_2_^−^) [17]. Under physiological conditions, O_2_^−^ is quickly removed, since it is converted by the mitochondrial-MnSOD into O_2_ and H_2_O_2_; hydrogen peroxide is then converted to water by catalase (CAT) and glutathione peroxidase (GPX) [144]. The imbalance in the SOD/GPX and CAT ratio occurring in DS cells prevents H_2_O_2_ scavenging, exacerbates cytosolic ROS accumulation and induces oxidative stress, which is particularly harmful and neuro-toxic in the brain (for refs., see [18]). The alteration of mitochondrial redox states associated with the overproduction of specific Hsa21 proteins, such as S100B and APP, has been proposed to promote cell injury and neuronal apoptosis [145].

D’Acunzo et al. have recently isolated and identified a new population of extracellular vesicles containing mitochondrial proteins, so-called “mitovesicles”, found to be altered in DS [146]. The authors provide evidence that these brain mitovesicles contain a specific subpopulation of mitochondrial components, and their levels and mitochondrial cargo are proven to be aberrant in DS. Comparative analysis of mitovesicles derived from the brains of Ts2 mouse models of DS and isolated from post-mortem human brains of individuals with DS revealed higher numbers of mitovesicles with altered composition in the DS brain parenchyma in both murine and human post-mortem brains [146]. These data suggest that mitochondrial impairment directly alters brain biology, either by triggering the release of these vesicles or by regulating the mitochondrial composition in the single extracellular vesicle.

On this basis, targeting mitochondria and/or signaling pathways controlling mitochondrial functions can have a great therapeutic potential in DS, since this can increase cell energy status and reduce oxidative stress. Therefore, many efforts have been performed to find therapeutic approaches that are able to attenuate mitochondrial dysfunction in DS.

Naturally occurring phytochemicals, and in particular polyphenols belonging to the class of flavonoids, have been deeply investigated as intriguing possibilities for the management of DS because of their multimodal actions in targeting and improving pathways, including regulatory genes and proteins of mitochondrial functions, disturbed by Hsa21 trisomy (see reviews [17,147,148,149]). Several flavonoids, including epigallocatechin-3 gallate (EGCG), resveratrol (RSV) and 7,8-dihydroxyflavone (7,8-DHF), have been shown both in vitro and in mouse models of DS to target Sirt 1/ PGC-1α/AMPK pathways [123,132,150], to modulate expression and activity of DYRK1A [151,152] and the cyclic AMP response-element binding protein (CREB) [153] and to potentially to regulate miR-155 [154], impacting mitochondria functions [16,17,18,147]. These polyphenols were able to fully rescue the activities of the defective MRC complex I and ATP synthase, increasing ATP production through OXPHOS, restoring the cellular and brain levels of ATP and decreasing mitochondrial ROS production and oxidative stress, with a consequent full restoration of hippocampal neurogenesis (for refs., see [132,147]). However, the main drawback in taking supplements containing polyphenols is that they have a limited bioavailability in humans. They have a quick metabolism, and substantial modifications take place during their systemic absorption or when dissolved in aqueous solutions, strongly impacting their efficacy [155,156]. For instance, a randomized, placebo-controlled phase 2 clinical trial demonstrated the efficacy of EGCG orally delivered in capsules (dose: 9 mg/kg per day) in improving the effects of cognitive training in young adults with DS [157]. The efficacy was not reproduced when, in a randomized phase 1 clinical trial, EGCG was supplemented at the same dose in children with DS in a powdered, chocolate-flavored formulation dissolved in 100 mL water [158]. Although this formulation has been proved to have a good bioavailability and absorption in the gastrointestinal tract in adults [159], EGCG modification in water cannot be excluded considering the fast degradation of EGCG in solutions if not immediately ingested. Actually, a lack of EGCG measured in plasma concentrations is indicated as a limitation of this study [158]. In two pilot clinical studies [160,161], we investigated the effect of the nutraceutical supplementation of EGCG in pediatric children, trying to improve EGCG delivery by creating an emulsion of the polyphenol (10 mg/kg/die) with fish oil omega-3, which was formerly proven to improve EGCG bioavailability [162]. In both studies, we demonstrate that nutraceutical supplementation can be safely administered in early childhood DS children, and this is able to revert the deficiency in the MRC complex I and ATP synthase. As regards the effects on cognitive functions in the two clinical studies, in the case study, a neuropsychological evaluation showed a significant improvement in the ability to perform tasks that require concentration [160]. However, in the pilot clinical study, the results on the improvement of cognitive performance were assessed as still inconclusive due to study limitations, such as a lack of placebo and the small number of DS children involved, especially when subgroups of age were considered [161].

In our opinion, the supplementation of pure decaffeinated EGCG, associated with other nutraceuticals that are shown to improve its bioavailability (see [147]), should be recommended in children and adults with trisomy 21 at a controlled dose under medical supervision, with the aim of improving mitochondrial energy deficits and to reduce oxidative stress.

The biguanide metformin, an FDA-approved drug used for the treatment of type 2 diabetes and for polycystic ovarium syndrome, has also been considered in the context of repurposed drug treatment in DS. Metformin is also able to counteract mitochondrial network impairment and correct mitochondrial dysfunction, which is shown in fibroblasts with Hsa21 trisomy, by activating PGC-1α/PPARGC1A signaling [163]. The same effect has been reported with pioglitazone [164], which belongs to thiazolidinediones that selectively stimulate PPAR-γ. It increases the phosphorylation of AMPK and, in turn, the expression of PGC-1α [165]. Actually, metformin and pioglitazone might be interesting drugs for DS, since they have been shown to promote neurogenesis and enhance spatial memory formation in adult mice [166], as well as to prevent cognitive impairment in neurodegenerative diseases [167]. However, prior to the clinical translation of metformin or pioglitazone as a chronic therapy in DS, clinical trials are needed to consider the relationship between dosage and effect, especially in children.

Other supplements that have long been studied in DS are the coenzyme Q10 (CoQ10) and folic acid; both are found to be reduced in children with DS [168,169]. CoQ10 is a crucial cofactor for the mitochondrial respiratory chain function, involved in free radical scavenging, whose supplementation in children with DS for a short time has been shown to induce protection against DNA oxidation by modulating DNA repair mechanisms [170]. When supplemented in those with DS, the mitochondrial folate pathway, recently shown to regulate the mitochondrial energy metabolism process [171], could reduce homocysteine levels found to be higher in some DS patients [169]. However, despite the promising data, clinical trials with chronic supplementation of either CoQ10 or folic/folinic acid have failed to improve biological or cognitive outcomes in early infancy of children with DS [172,173,174].

### 5.2. Rett Syndrome

Rett syndrome (RTT, OMIM #312750) is a rare genetic neurodevelopmental disease (the incidence is one in 10,000 live births) due, in most cases, to mutations in the X-linked gene encoding methyl-CpG-binding protein 2 (MeCP2) [175]. RTT, the second most prevalent cause of intellectual disability in the female gender, is characterized by a regression in neurodevelopment between 6 and 18 months of age that severely affects motor, cognitive and communication skills [176,177], with the development of autistic behavior as well [178]. MeCP2, a transcriptional repressor, causes chromatin compaction and gene silence by binding to methylated CG dinucleotides in specific gene promoters [179]. Sometimes it serves as a transcriptional activator [180] and also mediates splicing [181]. Although the condition is extremely complex, it seems clear that throughout its development, the MeCP2 mutation causes failure in the maturation of brain circuits, including neurons [182] and astrocytes [183], with reduced dendritic arborization. However, it is still unclear how mutations in MeCP2 lead to RTT’s symptomatology and its distinctive neuropathological symptoms. Given that RTT shares many clinical features with primary mitochondrial diseases (for instance, early symptom onset, delayed neurodevelopment, cognitive and motor regression), it has been long hypothesized that an energy metabolism impairment can occur early in RTT [9,184]. Indeed, mitochondrial phenotypes have been extensively studied in RTT in our laboratory and by other research groups, and convincing evidence for defective mitochondria in RTT has been found. A severe impairment of the OXPHOS apparatus, in particular a deficit of the MRC complex II and ATP synthase activities, has been shown in the brain of MeCP2-308 heterozygous female mouse models of RTT [16,19,185] and in cortical astrocytes [186], resulting in strong shortage of mitochondrial ATP production and a decrease in brain ATP levels; consistently, a large increase in ROS production by the defective complex II has been found [19]. The functional analyses performed in brain mitochondria isolated from MeCP2-Bird mice, another validated mouse model of RTT that bears a null MeCP2 mutation, revealed a much more affected bioenergetics of brain mitochondria. A severe deficit in complex I and II and ATP synthase activities was accompanied by a significant increase in ROS production from both complexes I and II [187]. The most severe profile of mitochondrial impairment found in MeCP2-Bird mice is consistent with the more severe behavioral phenotype presented by this mouse model of RTT [187]. These findings translate into redox state imbalance and oxidative damage in lipids and proteins, as described in fibroblasts from patients [188,189,190] and in brain and in astrocytes of murine models of RTT [191,192]. Ultrastructural changes have been shown in the cortical and hippocampal mitochondria of MeCP2^−/y^ mice [193] and confirmed in RTT patient fibroblasts [194]. Mitochondria appeared large and hyperfused, showing a loss of the physiological interplay between fusion and fission dynamics and the presence of defective dysregulation of mitochondrial quality control occurring through mitophagy and apoptosis [194]. Despite promising clinical trials [195], at present, there is no viable treatment for this very disabling disease. Therefore, improving mitochondrial dysfunction may be a useful therapeutic strategy that should be encouraged [196].

As for DS, we explored the repurposing of previously known drugs such as metformin as a pharmacological approach also in RTT. We demonstrate that symptomatic female RTT mouse models, which recapitulate symptoms of RTT, treated with metformin either systemically for 10 days long or chronically for 4 months resulted in the rescue of MRC complexes’ activities occurring with a PGC-1α-dependent mechanism [197,198]. In these studies, controls were used that excluded potential toxicity due to metformin treatment. Interestingly, modulation of PINK1, a mitophagy regulator, also occurs, possibly improving mitochondrial turnover, which is found to be impaired in RTT. This results in an improvement of mitochondrial OXPHOS and ATP brain levels with a concomitant decrease in ROS overproduction and oxidative stress [197,198]. The normalization of cognitive flexibility impairments in RTT female mice at an advanced stage of disease was also observed, suggesting that metformin may be a novel and adaptable therapy approach for RTT.

Interestingly, pharmacological systemic repeated stimulation of brain serotonin 7 receptor (5-HT7R) in different models of Rett syndrome [185,187] and the modulation of brain Rho GTPases by the bacterial toxin CNF1 [199] effectively restored mitochondrial impairments in RTT mouse models, resulting in a sustained phenotypic amelioration of the disease, possibly through the modulation of Rho GTPases and mTOR signaling [185,199].

Therefore, a direct or indirect correction of mitochondrial dysfunction in RTT is a potential therapeutic strategy that merits further exploration through a deep knowledge of the pathophysiological significance of the defective mitochondria, with the aim of identifying new possible targets for the development of novel pharmacological therapies for this disabling syndrome.

### 5.3. Fragile X Syndrome

Fragile X syndrome (FXS, OMIM #300624) is a genetic neurodevelopmental disorder characterized by a range of cognitive and behavioral deficits, including mild to moderate intellectual disability, and it is the leading monogenetic cause of autism [200,201]. Fragile X syndrome affects one in every 4000 males and one in 7000 females. Symptoms in males with FXS are more severe than in females, who may have normal intelligence to mild intellectual disability [202,203].

The genetic cause of FXS is the X-linked mutation of the *Fmr1* (fragile X mental retardation 1) gene. In the case of full mutation of *Fmr1*, i.e., when the expansion of a trinucleotide repeat of the CGG triplet within *Fmr1* exceeds 200, the gene is hypermethylated and is no longer able to code for the fragile X mental retardation protein (FMRP), whose expression is thus largely silenced [204]. *FMRP* is an RNA-binding protein, widely expressed in the brain, whose activity is essential for proper synaptic plasticity and the maintenance of neuronal synaptic connections [205].

Many studies have shown that the elimination of FMRP leads to significant morphological and functional changes within the brain, including abnormal synapse and circuitry development [206]. Of interest, a very recent study used FMRP knockout (KO) cells to demonstrate the importance of the interaction of FMRP with the voltage-dependent anion channel (VDAC) protein of the mitochondrial transition pore complex in regulating the formation of endoplasmic reticulum–mitochondria contact sites, calcium homeostasis and, in turn, synaptic formation and neuroplasticity [207].

An Fmr1 knockout (KO) mouse model that recapitulates several phenotypic aspects of the syndrome is crucial for understanding the pathogenesis of FXS and the genotype–phenotype correlation in the syndrome.

Fmr1-deficient mouse models develop symptomatology and histopathology consistent with human Fragile X disease, including abnormal synapse development [208] and functionally altered homeostatic neuronal plasticity [209]. Abnormal expression of mitochondrial genes, mitochondrial fragmentation and depolarization, aberrant mitochondrial function and increased oxidative stress are all signs associated with poor dendritic maturation and neuronal development impairment in Fmr1 KO mice [210].

We provided the first evidence of a defective and inadequate mitochondrial bioenergetics in the cerebral cortex of Fmr1 KO mice, sustaining the idea that dysfunctional mitochondrial energy metabolism may contribute to neurological impairment in FXS and be critically relevant in the pathogenesis of the syndrome [21]. A severe deficit in mitochondrial ATP production was found in both juvenile and adult Fmr1 KO mice, which is reflected in a deficit in cortical tissue energy status [21]. Surprisingly, despite the severe mitochondrial energy deficit, hyperactivation of all respiratory complexes as well as of the mG3PDH (mitochondrial glycerol 3-phosphate dehydrogenase) enzyme, a key component of the glycerophosphate shuttle that is particularly active in the brain, was measured in the cortex of both juvenile and adult Fmr1 KO mice. The aberrant mitochondrial bioenergetics and the age-dependent increase in ROS production detected in the Fmr1 KO mouse brain cortex [21] suggest that the mitochondrial abnormalities could favor an increase in oxidative stress in FXS (see Table 1). In this study, it has been also hypothesized that the hyperactivation of MRC complexes and the resulting inefficient oxidative phosphorylation in FXS could be a direct consequence of FMRP deficiency, since many mRNAs encoding for MRC complex subunits are direct FMRP targets [202,211].

In a recent work, Griffiths et al. investigated the functionality of forebrain mitochondria in Fmr1 KO mice during the stage of maximal synaptogenic activity by detecting low thermogenic oxygen consumption due to futile proton leakage in Fmr1 KO mitochondria as well as to reduced mitochondrial levels of coenzyme Q (CoQ) [212]. An external supply of CoQ, which replenishes the coenzyme mitochondrial pool, rescues the pathological proton leakage, restores protein synthesis rates during synaptogenesis and normalizes some key phenotypic features. The results are consistent and support the evidence that FMRP deficiency results in a severe deficit of OXPHOS efficiency during neurodevelopment, suggesting that dysfunctional mitochondria may contribute to the FXS phenotype. Licznerski et al. reported that Fmr1−/y mice treated with the ATP synthase modulator dexpramipexole showed improvement in some behavior valuations of autistic features [213].

**Table 1:** Neurobiological alterations and mitochondrial phenotypes in Down, Rett and Fragile X syndromes and drugs targeting mitochondria proved to improve neurobiology.

Genetic NDDs	Neurobiological Alterations	Mitochondrial Phenotype	Drugs Targeting Mitochondrial Signaling
Down syndrome	Defects in foetal and adult hippocampal neurogenesis; Reduced granule neurons and dendritic spine density; Impaired neuro-differentiation; Impaired neurotransmission; Axonogenesis and dendritic abnormalities; Astrocyte-mediated synaptic dysfunction; Reduced brain volume; Neurodegeneration. (For refs., see [16,17,18,124,125,126,127,128,129]).	Deficit in MRC complex I, ATP synthase, ANT and ADK activities [130,131]; Deficit in mitochondrial ATP production [131]; Reduced mitochondrial membrane potential [130]; Mitochondrial ROS overproduction [130]; Altered mitochondrial morphology, dynamics and biogenesis [127,134].	EGCG targets: DYRK1A; APP; cAMP/PKA; Sirt1/PGC-1α; AMPK [132,151,152] Resveratrol targets: RCAN1; miR-155; Sirt1/PGC-1α; AMPK [132,154] 7,8-DHF targets: TrkB receptor; MAPKs/CREB; PGC-1α [123] Metformin targets: NRIP1; PGC-1α/PPARGC1A [163]
Rett syndrome	Neurological cognitive and motor regression; Failure in the maturation of brain circuits; Reduced dendritic arborization; Reduced brain volume. (For refs., see [175,176,177,178]).	Down regulation of MRC complex II, complex III and complex IV expression [19,185]; Deficit in complex I, II and ATP synthase activity [19,187]. Reduced OXPHOS capacity [19,185,187]; Mitochondrial ROS overproduction [19,187]; Altered mitochondrial morphology and dynamics [193]; Reduced mitophagy [194].	Metformin targets: PGC-1α/TFAM; PGC-1α/NRF2; PINK1 [197,198] Bacterial Toxin CNF1 targets: Rho GTPases; mTOR. [185,197] LP-211 targets: 5-HT7R; Rho GTPases [187,199]
Fragile X syndrome	Abnormalities in dendritic spine shape; Altered neural differentiation and migration; Defective cortical maturation and synaptic plasticity. (For refs., see [200,201,202]).	Up regulation of MRC complexes expression and activities [21]; Reduced OXPHOS [21,213]; ROS overproduction [21]; Mitochondrial proton leak [212].	Mitochondrial CoQ target: Mitochondrial proton leak block [214] Dexpramipexole target: ATP synthase modulation [213]

In a recent study, a population of extracellular vesicles (EVs) carrying an affected mitochondrial cargo was isolated from astrocytes and cerebral cortices derived from Fmr1 KO mice by signaling their critical role as biomarkers of mitochondrial dysfunction in FXS [214]. Selective mitochondrial protein components, such as the transcription factor NRF-1 (nuclear respiratory factor 1) and the ATP5A and ATPB subunits of ATP synthase, were found to be drastically reduced both in EVs derived from cerebral cortices and in those secreted by the astrocytes of Fmr1 KO mice. This study suggests that the pathogenesis of FXS is strictly connected to mitochondrial dysfunction in astrocytes, which may be tracked by the depletion of mitochondrial components in EVs.

In their recent work, Bülow and coworkers gave evidence of dysregulated mitochondrial homeostatic plasticity that controls mitochondrial structure and polarity in dendrites and axons in Fmr1 KO murine models of FXS [215].

These results suggest that mitochondrial dysregulation in FXS might contribute to abnormal neuronal plasticity. Further investigations should explore the molecular mechanism regulating mitochondrial plasticity by checking the timing and causal link leading to mitochondrial alterations in FXS, as well as examining how the rescue of mitochondrial plasticity can in turn improve neuronal plasticity. Therefore, the restoration of mitochondrial function should be considered a conditio sine qua non for future therapeutic strategies. Mitochondria might be a potential new treatment target, not yet explored, for Fragile X syndrome.

## 6. Concluding Remarks

This review provides an updated overview of the emerging critical role of mitochondria as master regulators of brain bioenergetics in brain neurodevelopment and neuroplasticity, debating how bioenergetic dysfunction of mitochondria could be causally interrelated to the origins of a wide variety of childhood neurodevelopmental disorders.

We focus on Down, RTT and Fragile X syndromes, as these represent the most typical genetic syndromic conditions leading to intellectual disability. We remark upon how these disorders, though different in their genetic origin and aetiology, share significant clinical and neuropathological features (see Table 1) overlapping with brain energy deficits and defective mitochondrial bioenergetics, which are common pathogenesis factors in these diseases (Figure 2). We point to evidence that mitochondrial dysfunction may be a critical mechanism involved in the etiopathogenesis of intellectual disabilities and cognitive deficits, which represent key hallmarks of these NDDs.

Emerging insights into the mechanistic trends underlying neuropathological conditions are helping to change our point of view on the role of mitochondria and the timing of their involvement in these neurodevelopment disorders. Rather than the existence of a simple association and downstream effect with the pathogenetic events triggering syndromic conditions, mitochondrial dysfunction may be understood as an early and upstream causal event.

Here, we critically discuss how an improvement of mitochondrial function may attenuate cognitive impairment and a variety of other clinical sequels linked to NDD syndromes, improving overall health and the quality of life of patients with NNDs.

We believe it is suitable and indispensable to continue investigations aimed at deepening the molecular basis of these neurodevelopmental diseases. In this context, deciphering the role of mitochondria will allow us to shed light on critical pathways in the pathogenesis of these NNDs.

Interventions targeting mitochondria aimed at the maintenance of mitochondrial homeostasis could direct new therapeutic approaches in which mitochondria can become targets. In this way, we may be able to not only improve brain development, but also to provide support to the whole patient’s wellbeing from a holistic perspective.

## Data Availability

Not applicable.
